# Extracellular vesicle-microRNAs mediated response of bovine ovaries to seasonal environmental changes

**DOI:** 10.1186/s13048-023-01181-7

**Published:** 2023-05-23

**Authors:** Ahmed Gad, Kamryn Joyce, Nico Graham Menjivar, Daniella Heredia, Camila Santos Rojas, Dawit Tesfaye, Angela Gonella-Diaza

**Affiliations:** 1 Department of Biomedical Sciences, Animal Reproduction and Biotechnology Laboratory (ARBL), Department of Biomedical Sciences, Colorado State University, Fort Collins, CO USA; 2 Department of Animal Production, Faculty of Agriculture, Cairo University, Giza, 12613 Egypt; 3 North Florida Research and Education Center, Institute of Food and Agricultural Sciences, University of Florida, Marianna, FL USA

**Keywords:** Follicular fluid, Extracellular vesicles, miRNA, Heat stress, Beef cows

## Abstract

**Background:**

Among the various seasonal environmental changes, elevated ambient temperature during the summer season is a main cause of stress in dairy and beef cows, leading to impaired reproductive function and fertility. Follicular fluid extracellular vesicles (FF-EVs) play an important role in intrafollicular cellular communication by, in part, mediating the deleterious effects of heat stress (HS). Here we aimed to investigate the changes in FF-EV miRNA cargoes in beef cows in response to seasonal changes: summer (SUM) compared to the winter (WIN) season using high throughput sequencing of FF-EV-coupled miRNAs. In addition to their biological relevance, the potential mechanisms involved in the packaging and release of those miRNAs as a response to environmental HS were elucidated.

**Results:**

Sequencing analysis revealed that an average of 6.6% of the EV-RNA mapped reads were annotated to bovine miRNAs. Interestingly, miR-148a, miR-99a-5p, miR-10b, and miR-143 were the top four miRNAs in both groups accounting for approximately 52 and 62% of the total miRNA sequence reads in the SUM and WIN groups, respectively. A group of 16 miRNAs was up-regulated and 8 miRNAs were down-regulated in the SUM compared to the WIN group. Five DE-miRNAs (miR-10a, miR-10b, miR-26a, let-7f, and miR-1246) were among the top 20 expressed miRNA lists. Sequence motif analysis revealed the appearance of two specific motifs in 13 out of the 16 upregulated miRNAs under HS conditions. Both motifs were found to be potentially bonded by specific RNA binding proteins including Y-box binding proteins (YBX1 and YBX2) and RBM42.

**Conclusion:**

Our findings indicate that FF EV-coupled miRNA profile varies under seasonal changes. These miRNAs could be a good indicator of the cellular mechanism in mediating HS response and the potential interplay between miRNA motifs and RNA binding proteins can be one of the mechanisms governing the packaging and release of miRNAs via EVs to facilitate cellular survival.

**Supplementary Information:**

The online version contains supplementary material available at 10.1186/s13048-023-01181-7.

## Background

Global climate change directly impacts the livestock sector due to continued increases in atmospheric temperatures and as a result, animals are exposed to more adverse conditions of heat stress (HS). The global economic losses amid the dairy and beef industry due to HS are estimated to be between $14.89 – 39.94 billion per year by the end of the century [1]. This primarily stems from the significant impact of HS on animal productivity and the continued decline in fertility and reproductive performance [2]. The harmful effects of HS on ovarian functions and subsequent oocyte developmental competence are one of the main reasons driving reduced fertility [3]. The development of novel alternative strategies to mitigate the negative impacts of HS on reproductive function requires a more comprehensive understanding of heat-induced molecular alterations at the cellular level.

In bovine, follicular development is a lengthy process starting from the growth of primordial follicles to the preovulatory stage. Effects of maternal hyperthermia during specific stages of follicular growth and development may significantly affect follicular growth [4], in which the carryover effects of HS have the capacity to linger for months into the cool season resulting in long-term impacts on the reproductive performance of animals [5].

Seasonal effects on cows’ reproduction include different factors (e.g., feeding, daylight hours, etc.), however, the most important factor is the consequence of increased temperature and humidity that result in inadequate regulation of the cow’s body temperature, a reduction in appetite and dry matter intake, reduction in duration and intensity of estrus, as well as disruption in hormonal levels [6, 7]. Several seasonal studies have reported the sensitivity of the ovarian pool of oocytes to elevated temperatures expressed as reduced developmental competence [8, 9]. Seasonal HS is known to alter steroid production and the biochemical composition of the follicular fluid (FF). For instance, FF obtained from large follicles of cows during the hot season is evidenced by lowered steroid concentrations, reduced granulosa cell (GC) viability, and impaired aromatase activity [10]. Moreover, the biochemical changes in the FF of the dominant follicle from high-producing cows exposed to HS post-partum have been evidenced by reduced concentrations of glucose, IGF-1, and cholesterol [11]. At the cellular level, cells activate heat shock proteins (HSPs) and oxidative stress response machinery as a defensive mechanism in response to HS that could ultimately lead to cell apoptosis [12]. Activated HSPs, as well as RNA transcripts, could be released by stressed cells and uptaken by other recipient cells to modulate their immunological responses against stress [13–15]. In the intrafollicular microenvironment, such cellular communication is facilitated by the FF containing various paracrine factors important for cell–cell communication during follicular growth [16].

In the follicular microenvironment, one of the more recently discovered mechanisms that facilitate and modulate communicative measures between various cells and the oocyte is extracellular vesicles (EVs). EVs are nano-sized, membrane-bound, and evolutionarily conserved structures secreted from almost all cell types into the surrounding extracellular space and are preferentially found among almost all body fluids [17]. EVs are broadly categorized as exosomes, microvesicles, and apoptotic bodies according to their size and mode of biogenesis [18]. Their capacity to modulate intercellular crosstalk is largely through their ability to transfer various bioactive molecules, including mRNA, miRNAs, and proteins, between neighboring cells following secretion into body fluids [19]. We have recently shown that bovine GCs subjected to in vitro thermal stress release EVs harnessed with protective molecular signals that induce tolerance to recurrent thermal stress in naïve recipient cells [20]. In addition, those EVs were found to contain different miRNA profiles in response to HS. The emerging regulatory role of miRNAs in mediating the stress response in different species [21–24] sheds light on their use as potential biomarkers and/or tools to modulate the cellular stress response. Therefore, understanding the follicular responses, in terms of EV-coupled miRNAs, to HS in cows during the summer seasons will aid in determining the specific role of these miRNAs in mediating the HS response amid intrafollicular cellular communication. Here we aimed to investigate the follicular level response of beef cows to seasonal changes with regard to the FF-EV miRNA profiles. Moreover, miRNA motifs and the corresponding RNA binding proteins were identified and their potential involvement in the packaging and release of miRNAs into EVs as it relevantly correlates to cellular survival under HS conditions is elucidated.

## Materials and methods

### Animals and sample collection

The experiment was conducted at the UF/IFAS North Florida Research and Education Center (Marianna, Flora, USA). Eleven *Bos taurus* crossbreed open cows were included in the experiment after clinical and gynecological examinations. Transrectal ultrasound was conducted to evaluate ovaries and it was determined that all cows were cycling (bearing at least one CL or preovulatory follicle in one of the ovaries) and without any ovarian abnormalities (cysts, tumors, etc.) before each OPU session. The cows remain open, maintained in outdoor pens with bahia grass and fed bermudagrass hay to meet the nutritional requirements of mature cows. Also, cows had ad libitum access to water and mineralized salt during the whole study (from winter until summer). Body weight and Body condition score (scored from 1 (thin) to 9 (fat)) were not different between winter and summer (Winter: 580.2 ± 20.5 kg and BCS of 5.0 ± 0.0; Summer: 571.1 ± 18.1 kg and BCS of 5.0 ± 0.0; P > 0.05). Ovum pick-up (OPU) sessions were conducted by a single operator in two different seasons: Winter (January 2021) and Summer (August 2021). Before each OPU session cows were stimulated to increase the number of follicles. Briefly, on a random day of the estrus cycle (Day 0) a progesterone device (Eazi-Breed CIDR Cattle Insert; Pfizer Animal Health, New York, NY, USA) was inserted in the vagina and a single dose of a GnRH analog (Factrel, 2 mL, im, Pfizer Animal Health) was administrated. Follicular growth stimulation was conducted by giving 3 injections of FSH (Folltropin V, im, Vetoquinol, Bertinoro, Italy). On day 3, 3 ml (equivalent to 105 IU) of FSH were administrated in the morning and afternoon. On day 4, 2 ml of FSH were administrated in the morning (equivalent to 70 IU). Finally, on day 5, the CIDR was removed and OPU was conducted in the morning. Ultrasound examinations (Esaote ultrasound, MyLabDelta Vet, with 10–5 MHz transducer) were conducted to evaluate the presence of a corpus luteum and the number of follicles at days 0 and 5.

**Fig. 1:**
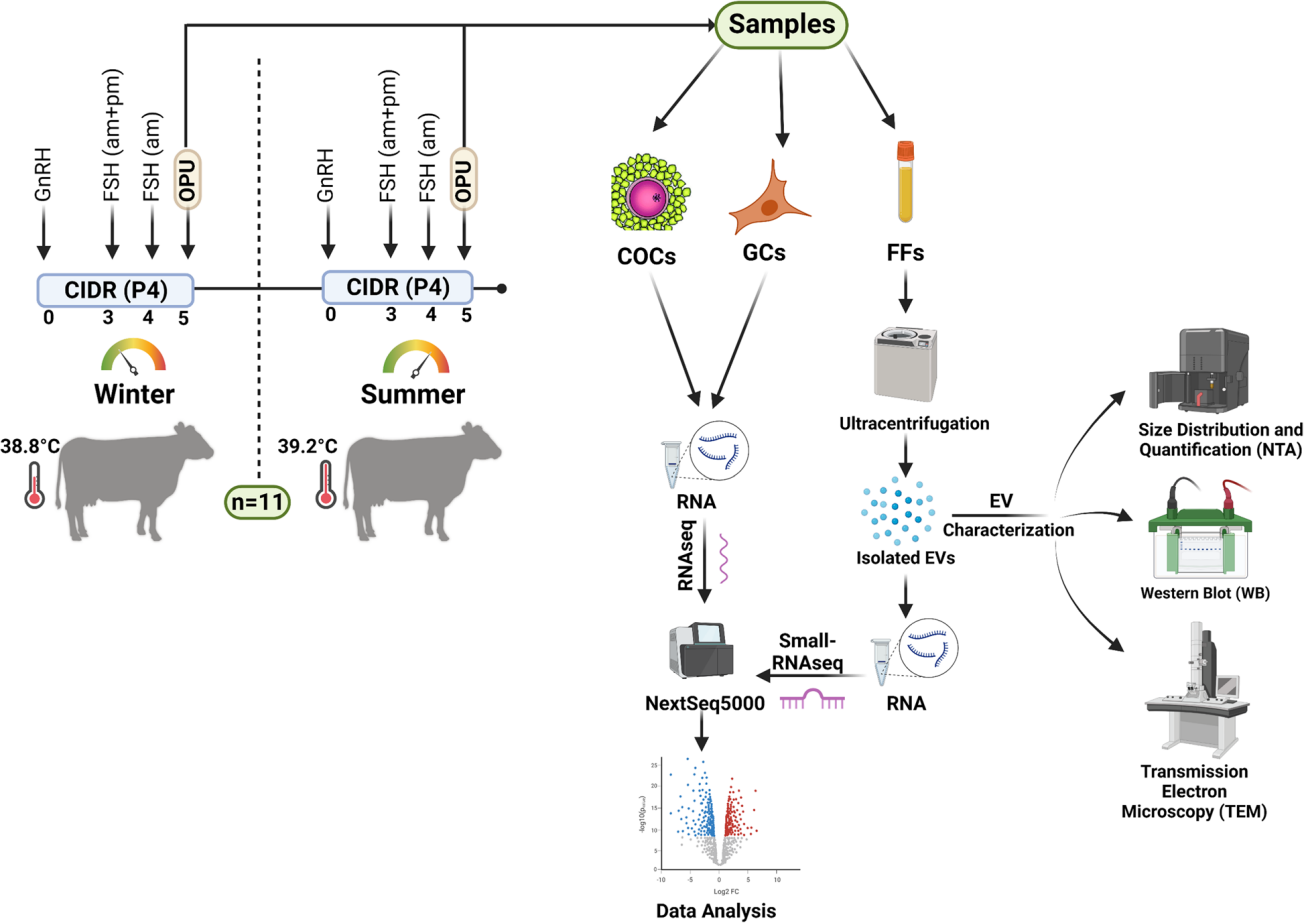
Experimental Design. Dry beef cows (*n* = 11) were subjected to synchronization and stimulation for follicular dynamics. Ovum pick-up (OPU) was conducted on all animals in the summer and winter seasons by ultrasound-guided transvaginal follicle aspiration. The follicular fluids (FFs) were collected from each target group (4 biological replicates/group; 8 mL FF/replicate). EVs were then isolated from the FFs using high-speed ultracentrifugation. Isolated EVs were characterized using NanoSight Tracking Analysis, western blot for EV and cellular marker proteins, and transmission electron microscopy. Total RNA was isolated from FF-EVs and small-RNA library preparation and RNAseq (NextSeq500; Illumina) were performed

OPU was performed using a real-time B-mode ultrasound scanner (Mindray 2200; Mindray Bio-Medical Electronics, Shenzhen, China) equipped with a 5-MHz micro-convex transducer (Mindray model 65C15EAV, Mindray Bio-Medical Electronics, Shenzhen, China) and coupled to a follicular aspiration guide (WTA, São Paulo, Brazil) and a stainless-steel guide. The follicular puncture was performed using a disposable 18 G hypodermic needle connected to a 50-mL conical tube via a suitable silicon tubing system (WTA). The pressure for aspiration was maintained using a vacuum pump (WTA model BV-003, WTA) with negative pressure adjusted between 60 and 80 mmHg. After the OPU of both ovaries, the aspiration system was replaced with a new one before conducting OPU for the next cow. The complete experimental design is presented in Fig. 1.

### Summer vs. winter environmental conditions

As previously stated, OPU was conducted on the same animals in two different seasons. Environmental data (average minimum and maximum temperature and relative humidity) from the 3 weeks before each OPU session was collected using the Florida Automated Weather Network (FAWN; https://fawn.ifas.ufl.edu/data/reports/). For the winter OPU, data were collected from February 2 to 23 of 2021, and for the summer OPU, data were collected from July 23 to August 13 of 2021. Next, the temperature humidity index (THI) was calculated using the following equation: THI = (1.8 × T + 32)—[(0.55—0.0055 × RH) × (1.8 × T—26)], where T = air temperature (°C) and RH = relative humidity (%) [25]. As expected, average temperature (summer: 27.48 °C; winter: 11.46 °C), minimum temperature (summer: 23.14 °C; winter: 6.45 °C), maximum temperature (summer: 33.66 °C; winter: 16.14 °C), and relative humidity (summer: 82%; winter: 83%) were contrasting in the summer compared with the winter. Also, when estimating the THI, the average THI in the summer was 79 while in the winter was 53. In Bos taurus beef cattle, a THI equivalent to 75 is an indicator of heat stress [26, 27]. Three weeks before the summer OPU session THI was always over 75, ranging from 75.47 to 82.45.

### Isolation of extracellular vesicles from follicular fluid

Four different FF samples (pool of two animals in each) from each group were used for EV isolation. Follicular fluid samples were subjected to a series of centrifugations starting at 500 xg for 10 min to remove cells, followed by 3000 xg for 10 min to remove the cellular debris, and at 12,000 xg for 30 min to remove protein aggregates and large particles. All centrifugation steps were performed at 4 °C. The supernatant of FF samples was filtered through a 0.22 μM sterile filter to remove particles larger than 200 nm. For the EV isolation, 2 ml of pre-centrifuged follicular samples were subjected to an ultracentrifugation procedure at 120,000 xg for 70 min at 4 ^O^C using the Beckman SWTi55 rotor. The EVs pellet was washed with sterile PBS and then centrifuged again at 120,000 xg for 70 min. Finally, EVs were resuspended in 500 μL of PBS and stored at − 80 °C until further characterization and analysis.

### Morphological and molecular characterization of EVs

The presence of EV-specific proteins (CD63, TSG101, FLOT1) in the isolated EVs and the absence of a cell-specific marker protein, cytochrome C (CYCS), were verified by the immunoblotting technique as we previously described [20]. Briefly, 100 μL of isolated EVs were lysed in 50 μL 1 × RIPA buffer and the protein extract samples were centrifuged at 12,000 × *g* for 30 min at 4 °C. Following this, protein lysates were separated in 10–12% gradient SDS-PAGE gel (Bio-Rad Laboratories, USA) at 90 V for 15 min and 125 V for 60 min and transferred onto a nitrocellulose membrane (Bio-Rad Laboratories, USA) for 1 h at 100 V. Membranes were blocked in 5% non-fat dry milk dissolved in TBST for 1 h on a shaker at room temperature. Following blocking, membranes were incubated with anti-CD63 rabbit polyclonal (1:250 System Biosciences, USA), Anti-TSG101 rabbit polyclonal (1:250 System Biosciences, USA), Anti-FLOT1 rabbit polyclonal (1:250 System Biosciences, USA), and Anti-CYTc goat polyclonal (1:350 Santa Cruz Biotechnology, Germany) primary antibodies overnight at 4^0^C. After washing the membranes with 1 × TBST, membranes were incubated with appropriate secondary antibodies conjugated with horseradish peroxidase for 1 h at room temperature protected from light. After washing the membranes, protein bands were visualized using an enhanced chemiluminescence substrate (Bio-Rad Laboratories, USA) and images were acquired using Chemi Doc XRS + chemiluminescence imaging system (Bio-Rad Laboratories, USA).

The morphology of the purified EVs were analyzed using a transmission electron microscope (TEM) according to the methods previously reported [20]. Briefly, a drop of 30 μl purified EVs was placed on parafilm. The EVs drops were covered with Formvar/carbon-coated grids and allowed to stand for 5 min to absorb the EVs. The grids that contained the EVs were washed with drops of ddH_2_O and fixed by placing them on a 30 μL drop of 2% uranyl acetate. The presence of the EVs on the carbon-coated grids was examined under an electron microscope. TEM imaging was done on a FEI/TFS Tecnai T12 Spirit TEM (FEI Company; Hillsboro, OR, USA), operating at 100 kV, with an AMT CCD.

The concentration and size distribution of isolated EVs were determined using the Zetaview Particle Metrix (Particle Metrix, Germany). Briefly, 10 μl of purified EVs was diluted in 990 μl of sterile PBS and assembled into the Zetaview Laser scattering microscope (Particle Metrix, Germany) fitted with an LM14C laser. For each sample, 11 independent video measurements were recorded at 11 independent positions, and video files were analyzed with ZetaView software version 8.05.12. All experimental parameters related to EV isolation and characterization have been submitted to the EV-TRACK knowledgebase (https://evtrack.org) under the EV-TRACK ID EV220402.

### Total RNA extraction, library preparation, and sequencing

Total RNA including miRNAs was isolated from EVs using a Norgen Exosomal RNA Isolation kit (Norgen, Canada), according to the manufacturer’s instructions. On-column DNA digestion was performed to remove genomic DNA contaminants. The RNA concentration and size distribution were analyzed using an Agilent RNA 6000 Pico kit in an Agilent 2100 Bioanalyzer (Agilent Technologies, Santa Clara, CA, USA). Small-RNA libraries were prepared for next-generation sequencing (NGS) using a TruSeq Small RNA Library Prep Kit (Illumina) according to the manufacturer’s instructions. Library quantity and quality assessments were performed using a Qubit DNA HS Assay Kit in a Qubit 2.0 Fluorometer (Thermo Fisher Scientific) and an Agilent DNA High Sensitivity kit in an Agilent 2100 Bioanalyzer (Agilent Technologies), respectively. The precise concentration of the libraries was calculated using a quantitative PCR. The libraries were pooled in equimolar ratios and then sequenced in a NovaSeq6000 sequencing instrument (Illumina, Inc., San Diego, CA, USA) as single-end reads (50 bases).

### Sequencing data analysis

FASTQ files were generated for each sample using the software bcl2fastq (Illumina Inc., San Diego, CA), and their quality was checked using the FastQC tool version 0.11.9. Data were analyzed using the software CLC Genomics Workbench, version 21. Raw sequence reads were trimmed based on quality score (Q-score > 30), ambiguous nucleotides (maximum two nucleotides allowed), read length (≥ 15 nucleotides), and adapter sequences were also removed. Reads were mapped to the bovine reference genome (ARS-UCD1.2) and annotated against bovine precursor and mature miRNAs listed in the miRBase database (release 22) using the CLC Genomics Workbench RNA-Seq Analysis and Quantify miRNA tools, respectively, applying the default software parameters. Raw expression data were normalized using the trimmed mean of M-values normalization method (TMM normalization) [28] and presented as TMM-adjusted Counts Per Million (CPM). The CLC Genomics Workbench Differential Expression tool was used for the expression analysis comparison of the two groups. MiRNAs with fold change (FC) > 1.5, p-adjusted value (FDR < 0.1 [29]), and average CPM > 10 were considered differentially expressed (DE). The raw FASTQ files and processed CSV files have been deposited in the NCBI’s Gene Expression Omnibus (GEO) with the accession number GSE221198.

### qRT-PCR validation

To validate the miRNA sequencing data, five DE-miRNAs (three from SUM upregulated and two from downregulated), as representative candidates, were selected for expression validation using qRT-PCR. For this, three independent EV samples from three different animals were used for total RNA including miRNAs isolation as mentioned above, and selected miRNAs were quantified using specific TaqMan miRNA Assays (Applied Biosystems, Foster City, CA, USA) using a real-time PCR (Bio-Rad Inc.) according to the manufacturer’s instructions. In brief, for each miRNA, a total of 5 ng RNA was reverse transcribed (RT) using a TaqMan microRNA Reverse Transcription Kit (Thermo Fisher Scientific, Waltham, MA) and miRNA-specific stem-loop primer (Applied Biosystems). RT reaction mixtures were incubated at 16 °C then 42 °C for 30 min each, followed by 85 °C for 5 min. The qRT-PCR was conducted in a 20 μl reaction mixture containing 2 μl cDNA sample, 1 μl FAM-labeled TaqMan assay, 10 μl TaqMan Universal PCR Master Mix (Applied Biosystems), and 7 μl nuclease-free water. Reaction conditions were as follows: initial denaturation at 95 °C for 10 min, followed by 40 cycles consisting of denaturation at 95 °C for 15 s, annealing, and extension at 60 °C for 60 s. Expression values were normalized to the geometric mean of miR-125 and miR-191 expression levels, the most stably expressed miRNA across all samples based on the NormFinder analysis. Statistical analysis of miRNA expression data was performed using Student’s t-test and statistical significance was identified at *P* ≤ 0.05.

### Target gene prediction and ontological classification

Genes targeted by the DE-miRNA were identified using the human homologous miRNAs in the miRWalk database [30] to enhance the target prediction. Within the miRWalk, validated target genes from miRTarBase (version 7.0) and commonly target genes predicted by TargetScan (version 7.1) and miRDB (release 5.0) were selected for ontological classification analysis using the DAVID bioinformatics web tool (https://david.abcc.ncifcrf.gov/). Pathways and biological processes (BP) were determined from the KEGG pathway database [31], and GOTERM_BP_DIRECT annotation set, respectively. Terms with low gene count (< 5 genes) were filtered out from the pathways and BP lists. The interaction networks of the targeted genes and the identified pathways were constructed with Cytoscape [32].

### Sequence motif analysis

Sequence-specific miRNA motifs (4–6 base) that are enriched in the up-regulated miRNAs compared to the down-regulated miRNAs (control sequences) were identified using Multiple Expectation Maximization for Motif Elicitation (MEME) suite v. 5.5.0 [33]. Motifs that commonly appeared in at least 50% of the submitted miRNAs were selected for further analysis. To determine the motif-associated RNA binding proteins (RBPs), the selected motifs were submitted to the TOMTOM motif comparison tool [34] using the RNA database.

## Results

### Identification and characterizations of isolated EVs

**Fig. 2:**
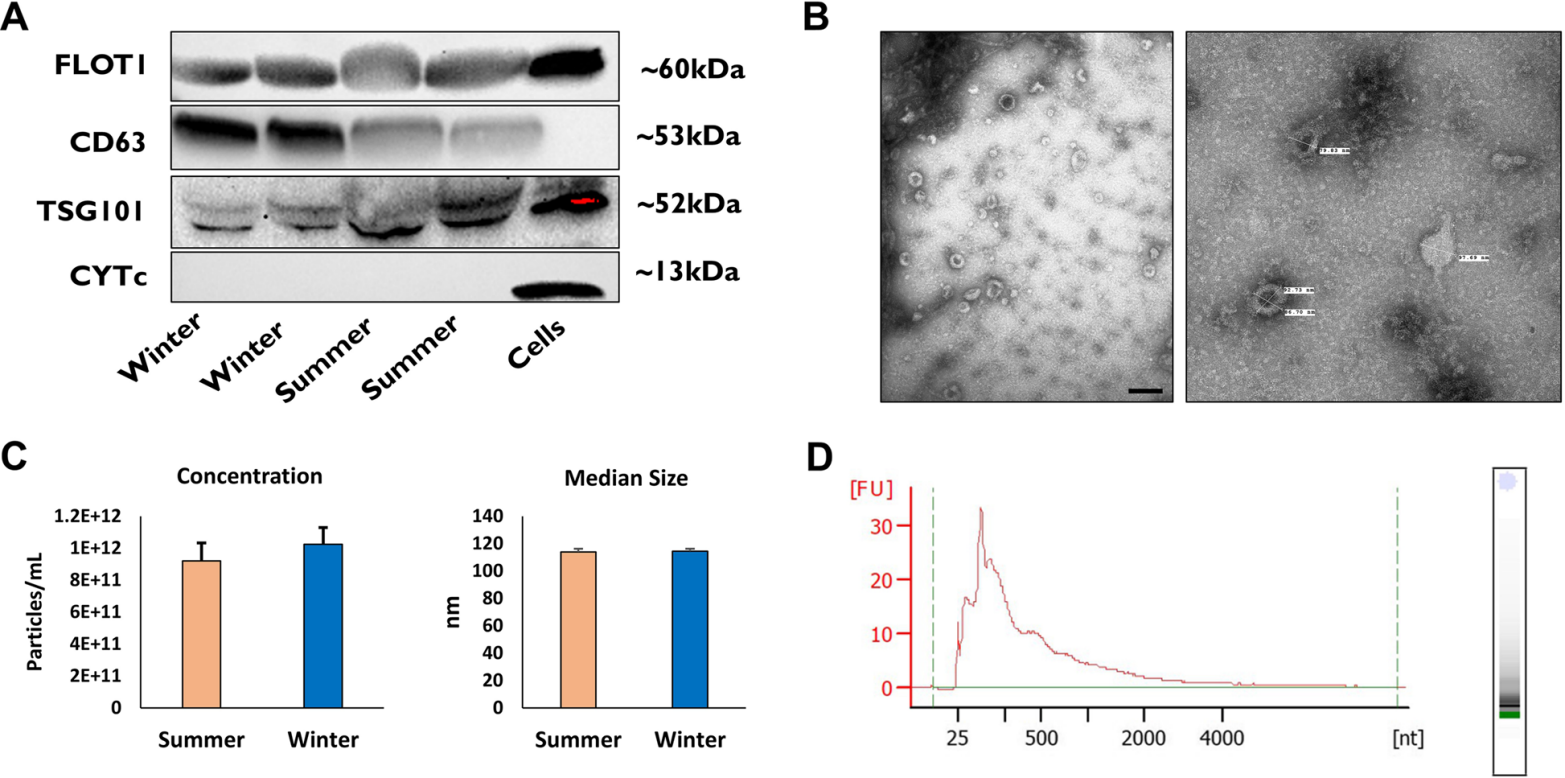
Morphological and molecular characterization of EVs. Western blot analysis of EVs marker proteins FLOT1, CD63, and TSG101 and cellular protein contamination indicator CYTc **(A)**. Transmission electron microscope (TEM) imaging shows a clear morphology of EVs with a cross-sectional size of the diameter, scale bar = 200 nm **(B)**. The concentration and median size of the isolated EVs using the NTA analysis **(C)**. The RNA size distribution of EVs shows the presence of the peak of small RNA and the absence of the 18 s and 28 s ribosomal RNA peaks **(D)**

Follicular fluid-derived EVs from SUM and WIN groups were characterized morphologically and molecularly according to the recommendations set by the International Society of Extracellular Vesicles (ISEV) [35]. The western blot analysis showed that the FF-EVs from both groups are enriched with the transmembrane proteins FLOT1, CD63, and TSG101 protein. Moreover, the mitochondrial protein marker, Cytochrome C was absent in the EV samples, but present in bovine GCs (Fig. 2A), confirming the purity of the EVs and the absence of cellular contaminants in the EV preparation. TEM imaging showed the presence of EVs with visible bilipid membranes within the acceptable size ranges (Fig. 2B). The NTA analysis showed that the concentration of EV samples from both groups was within the range of 3.5 × 10^11^ to 1.75 × 10^12^ particles/mL with no significant differences between the groups. Similarly, the EVs median size was around 114 nm in both groups with no significant differences (Fig. 2C). The total RNA isolated from the EV samples were analyzed for their integrity and the electropherogram analysis showed a clear peak for the small RNA size ranges and the absence of rRNA subunit peaks (18 s and 28 s) which characterize the cellular RNA, indicating no cellular contamination in the isolated EV samples (Fig. 2E).

### MiRNA expression profiles and differential expression analysis

**Fig. 3:**
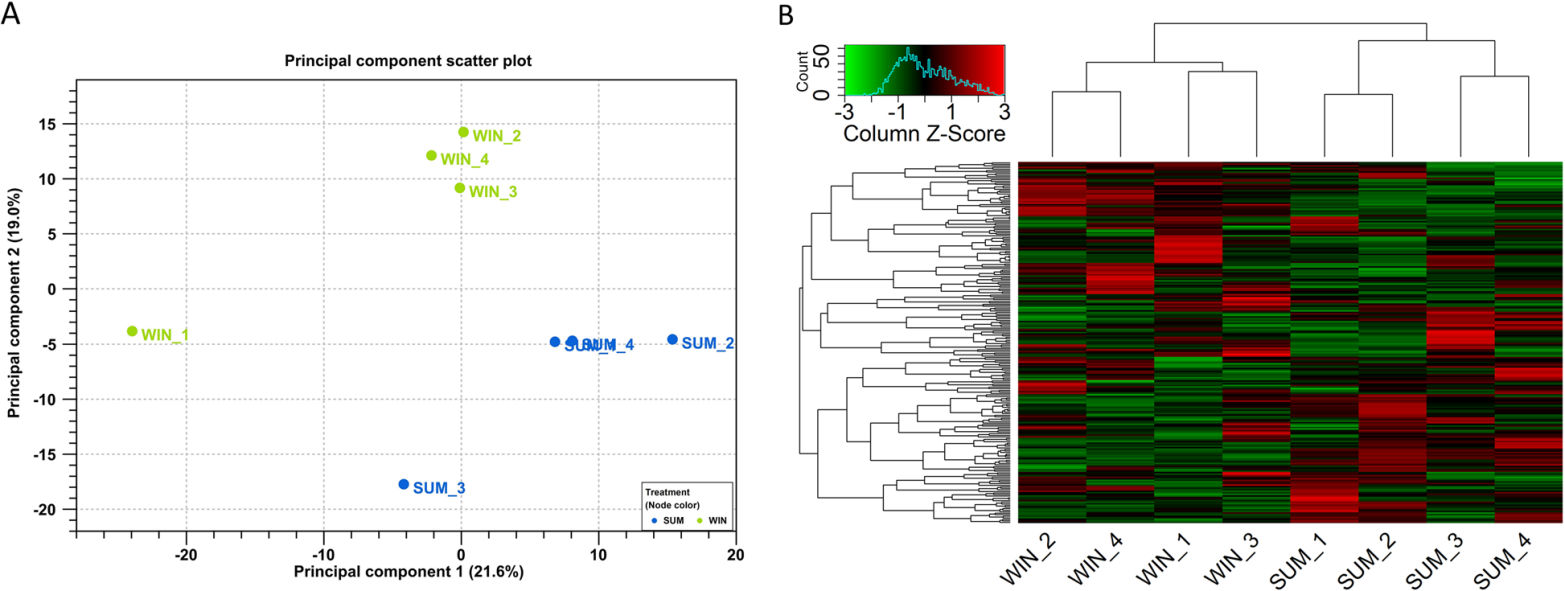
Small-RNA sequence data overview. Principal Component Analysis (**A**). Heatmap and hierarchical clustering of expressed miRNAs. Red and green colors represent high and low expressed miRNAs, respectively **(B)**. SUM: summer group; WIN: winter group

**Fig. 4:**
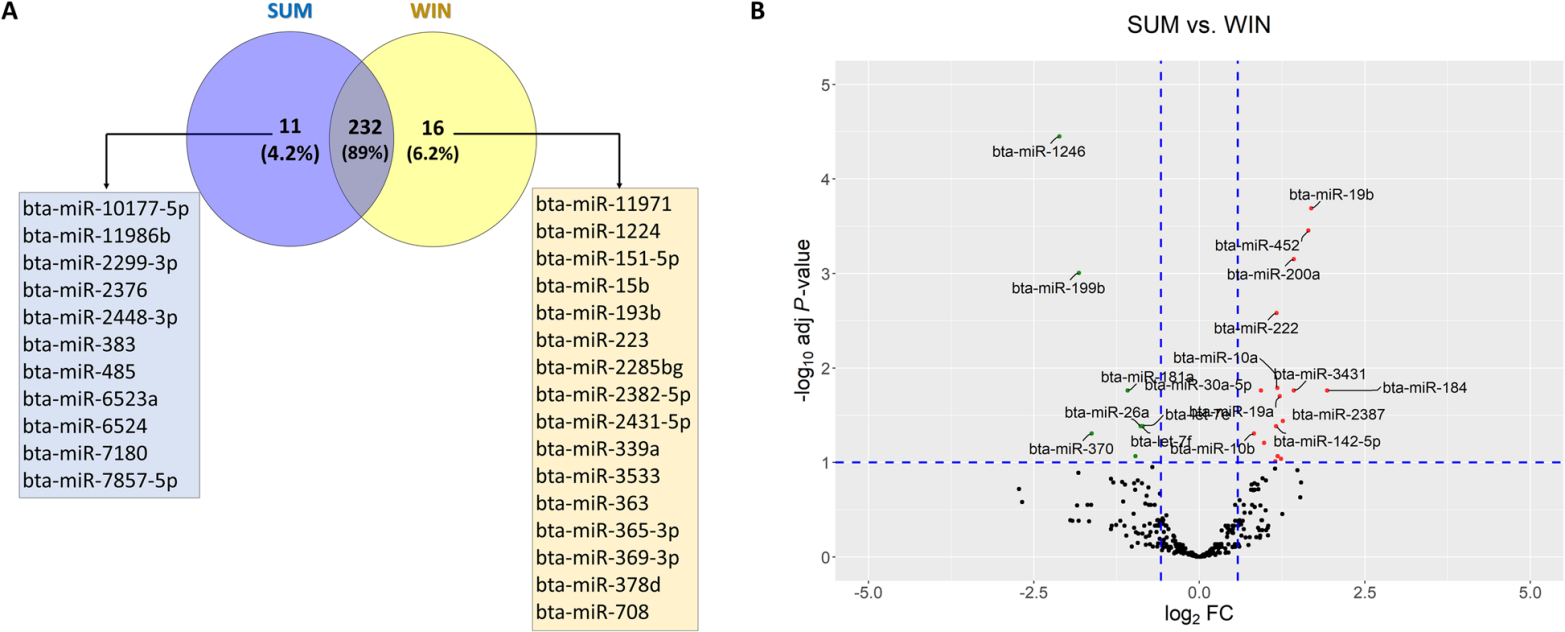
Differentially expressed miRNAs. Venn diagram for commonly and exclusively expressed miRNAs in the FF-EVs of summer (SUM) and winter (WIN) groups **(A)**. Volcano plot of expressed miRNAs. Up- and downregulated miRNAs in the SUM compared to the WIN FF-EV groups are labeled with red and green points, respectively **(B)**

A total of eight small-RNA libraries were constructed with approximately 20 million reads per library that passed the QC parameters with an average of 85% mapped to the bovine reference genome. Out of the mapped reads, an average of 6.6% were annotated to the bovine miRNAs from the miRBase database (Supplementary Table S1). Based on the miRNA expression profiles, principal component analysis (PCA) and heatmap exhibited a clear clustering of the replicates of each group (Fig. 3). A total of 243 and 248 miRNAs were considered as expressed (> 10 CPM) in the SUM and WIN groups, respectively, with 232 miRNAs being expressed in common and a total of 11 and 16 miRNAs were found to be exclusively detected in SUM and WIN groups, respectively (Fig. 4A). The top 20 highly expressed miRNAs are presented in Table 1 and the complete list of all expressed miRNAs is presented in Supplementary Table S2. Among the top 20 expressed miRNAs, 18 miRNAs were commonly detected in both groups. Interestingly, miR-148a, miR-99a-5p, miR-10b, and miR-143 were the top four miRNAs in both groups and accounting for approximately 52 and 62% of the total miRNA sequence reads in the SUM and WIN groups, respectively (Table 1).

**Table 1:** List of top 20 most abundant miRNAs in the extracellular vesicles obtained from follicular fluids from SUM and WIN groups

Name	SUM	Name	WIN
bta-miR-148a	335,249	bta-miR-148a	265,292.7
bta-miR-99a-5p	146,729.6	bta-miR-99a-5p	100,948.9
bta-miR-10b	131,675.5	bta-miR-10b	74,287.82
bta-miR-143	48,985.58	bta-miR-143	46,764.92
bta-let-7b	29,996.77	bta-miR-26a	43,106.6
bta-miR-10a	28,665.65	bta-let-7a-5p	35,540.64
bta-miR-26a	23,293.75	bta-miR-1246	35,452.23
bta-let-7a-5p	21,727.87	bta-let-7b	28,978.75
bta-miR-320a	20,767.93	bta-let-7i	23,237.47
bta-miR-21-5p	15,787.62	bta-miR-320a	22,398.35
bta-let-7i	15,370.83	bta-let-7f	21,824.77
bta-miR-151-3p	13,991.34	bta-let-7c	15,041.31
bta-miR-27b	12,268.99	bta-miR-21-5p	14,597.74
bta-miR-128	11,920.15	bta-miR-10a	12,673.24
bta-let-7f	11,891.01	bta-miR-423-5p	10,828.92
bta-miR-25	11,251.66	bta-miR-151-3p	10,629.91
bta-miR-378	10,807.93	bta-miR-146b	10,133.07
bta-let-7c	10,641.53	bta-miR-128	9588.558
bta-miR-423-5p	10,115.64	bta-let-7 g	9309.91
bta-miR-146b	9761.571	bta-miR-27b	9140.885

The expression values indicated as the mean of TMM-adjusted Counts Per Million (CPM)

Differential expression analysis indicated a total of 24 miRNAs as significantly differentially expressed between the two groups (FC > 1.5, FDR < 0.1, CPM > 10**)**. A group of 16 miRNAs was up-regulated and 8 miRNAs were down-regulated in the SUM compared to the WIN group (Table 2 and Fig. 4B). MiR-184, miR-19b, and miR-452 were up-regulated and miR-1246, miR-199b, and miR-370 were downregulated with more than three folds in the SUM compared to the WIN group. Interestingly, five DE-miRNAs (miR-10a, miR-10b, miR-26a, let-7f, and miR-1246) were also among the top 20 expressed miRNA lists.

**Table 2:** Differentially expressed (DE) miRNAs in extracellular vesicles obtained from follicular fluids of SUM compared to WIN group

Name	Sequence	FC	FDR
bta-miR-184	TGGACGGAGAACTGATAAGGGT	3.81	0.017351
bta-miR-19b	TGTGCAAATCCATGCAAAACTGA	3.23	0.000205
bta-miR-452	TGTTTGCAGAGGAAACTGAGAC	3.14	0.000351
bta-miR-200a	TAACACTGTCTGGTAACGATGTT	2.69	0.000704
bta-miR-3431	CCTCAGTCAGCCTTGTGGATGT	2.69	0.017329
bta-miR-2387	TGGAAGGCCTGGCTTTGCAGCG	2.40	0.036309
bta-miR-2408	CACGTGTGTGAGCTCAGCCGG	2.35	0.091029
bta-miR-19a	TGTGCAAATCTATGCAAAACTGA	2.32	0.019879
bta-miR-95	TTCAACGGGTATTTATTGAGCA	2.27	0.085596
bta-miR-10a	TACCCTGTAGATCCGAATTTGTG	2.26	0.0162
bta-miR-222	AGCTACATCTGGCTACTGGGT	2.25	0.00262
bta-miR-142-5p	CATAAAGTAGAAAGCACTAC	2.23	0.041243
bta-miR-24-3p	TGGCTCAGTTCAGCAGGAACAG	2.21	0.097153
bta-miR-2483-3p	AAACATCTGGTTGGTTGAGAGA	1.97	0.062057
bta-miR-30a-5p	TGTAAACATCCTCGACTGGAAGCT	1.91	0.017351
bta-miR-10b	TACCCTGTAGAACCGAATTTGTG	1.77	0.049396
bta-let-7e	TGAGGTAGGAGGTTGTATAGT	-1.81	0.041243
bta-let-7f	TGAGGTAGTAGATTGTATAGTT	-1.83	0.041243
bta-miR-26a	TTCAAGTAATCCAGGATAGGCT	-1.85	0.041243
bta-miR-126-3p	CGTACCGTGAGTAATAATGCG	-1.95	0.085596
bta-miR-181a	AACATTCAACGCTGTCGGTGAGTT	-2.12	0.017351
bta-miR-370	GCCTGCTGGGGTGGAACCTGGT	-3.09	0.049396
bta-miR-199b	CCCAGTGTTTAGACTATCTGTTC	-3.53	0.000985
bta-miR-1246	AATGGATTTTTGGAGCAGG	-4.33	3.54E-05

*FC* Fold Change, *FDR* False Discovery Rate

### qRT-PCR validation

**Fig. 5:**
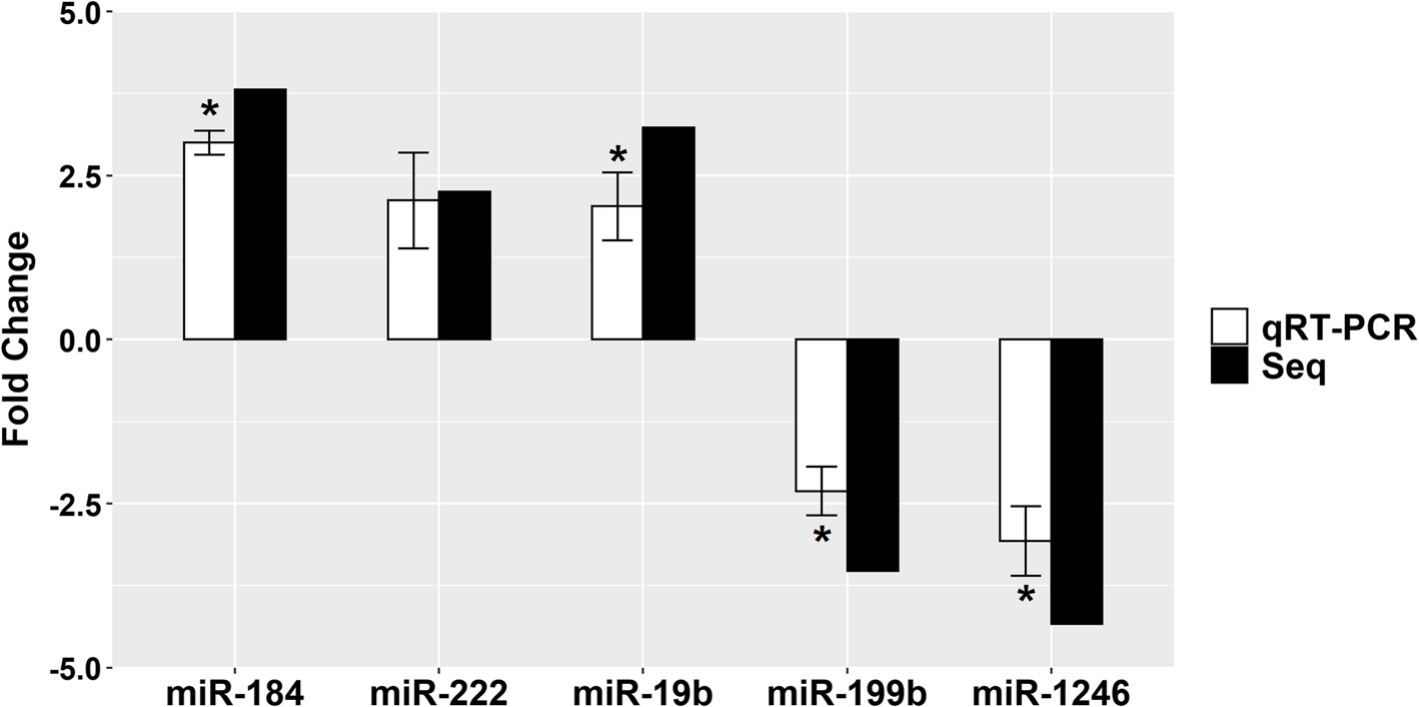
qRT-PCR analysis. Expression validation of the selected DE-miRNAs in comparison to the RNAseq (Seq) analysis. *Statistical significance between the summer and winter FF-EV groups (*P* < 0.05)

To validate the sequencing data, a group of 5 DE-miRNAs was selected and quantified using qRT-PCR. All miRNAs exhibited the same expression pattern as in the miRNAseq data (*P* < 0.05) except for miR-222, which showed the same pattern but with no statistical significance between the two groups (Fig. 5).

### Target gene prediction and gene ontology

**Fig. 6:**
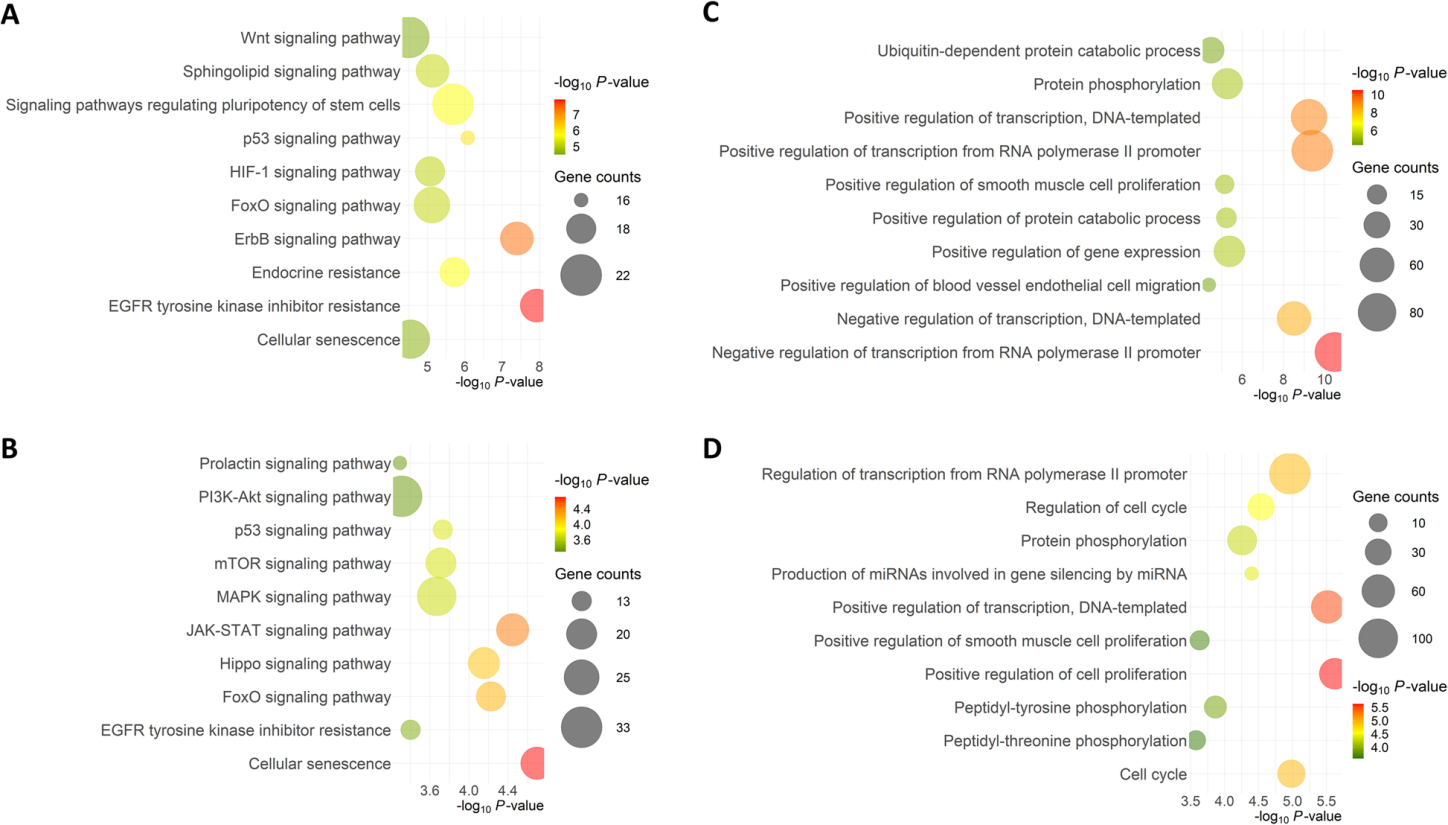
Ontological Classification. Top 10 pathways and biological processes targeted by the up- **(A** and **C**, respectively**)** and down-regulated miRNAs (**B** and **D**, respectively**)** in the summer compared to the winter FF-EV groups

**Fig. 7:**
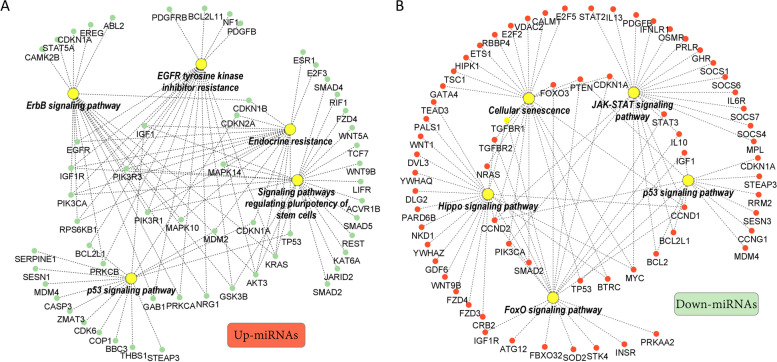
Interaction networking. The top 5 pathways and their corresponding genes targeted by up **(A)** or downregulated **(B)** miRNAs in the summer compared to the winter group

Target gene analysis revealed a total of 871 and 909 genes as potential targets of up and downregulated miRNAs, in SUM group respectively, with 140 genes commonly targeted by both groups of miRNAs. Ontological classification of these genes showed that EGFR tyrosine kinase inhibitor resistance, ErbB signaling, p53 signaling, and endocrine resistance were the top significant pathways targeted by the upregulated miRNAs in the SUM compared to the WIN group. On the other hand, cellular senescence, JAK-STAT signaling, FoxO signaling, and hippo signaling were the top significant pathways targeted by the downregulated miRNAs (Fig. 6, Supplementary Table S3). Regulation of transcription and gene expression were the top significant biological processes targeted by the elevated miRNAs in the SUM compared to the WIN group while regulation of cell proliferation and cell cycle were the top significant biological processes targeted by the downregulated miRNAs (Fig. 6, Supplementary Table S4). The interaction networks of the top 5 pathways and their corresponding genes targeted by the DE miRNAs are presented in Fig. 7.

### miRNA sequence motif analysis

**Fig. 8:**
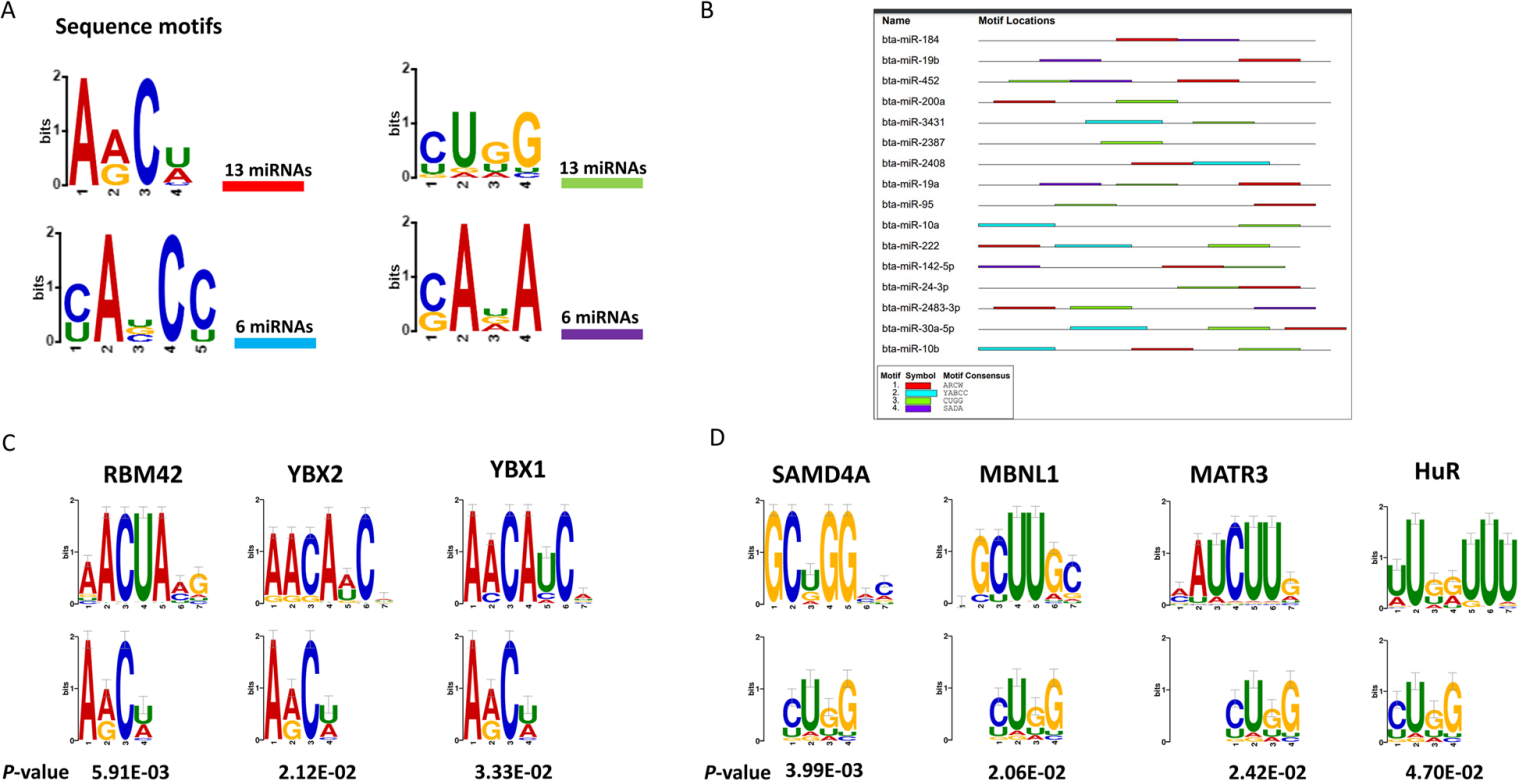
miRNA sequence motif analysis. Four identified motifs in different numbers of the up-regulated miRNAs in the summer compared to the winter group **(A)**. The locations and distribution of the identified motifs on the up-regulated miRNAs **(B)**. The alignments of the AACU **(C)** and CUGG **(D)** motifs with the known RNA binding protein motifs (*P* < 0.05)

To identify potential sequence motifs and the corresponding RNA binding proteins associated with the regulation of the packaging and release of the candidate miRNAs into EVs in response to thermal stress, we performed motif sequence analysis of the miRNAs that were enriched in SUM FF-EVs compared to the miRNAs that were enriched in WIN FF-EVs group. We identified four different motifs (4–5 bases). Two of them significantly appeared on more than 80% (13 out of 16) of the up-regulated miRNAs (Fig. 8A and B; Table 3). RBP-specific motif matching showed that the first motif (AACU) is potentially targeted by RBM42, YBX2, and YBX1 proteins (Fig. 8C) while the second motif (CUGG) is potentially targeted by SAMD4A, MBNL1, MATR3, and HuR proteins (Fig. 8D).

**Table 3:**
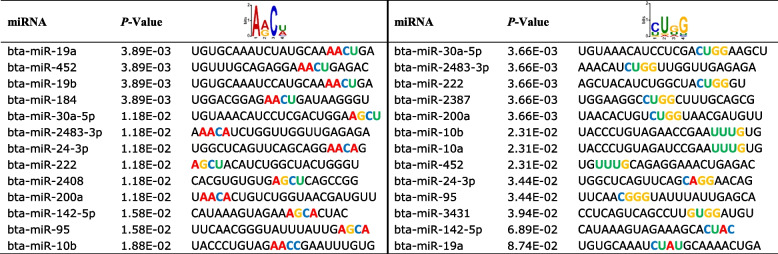
The identified motif positions on the up-regulated miRNAs in the SUM compared to the WIN group

## Discussion

Extracellular vesicles are known to shuttle ample bioactive molecules (mRNA, miRNA, proteins, and lipids) reflecting the physiological status of the secreting cells, [36, 37] leading to alterations in gene expression and function in recipient cells [38]. Our previous studies indicated that the bovine FF-EVs-miRNA profiles are associated with oocyte developmental competence [39] and the post-calving metabolic status of dairy cows [40]. We have also reported that, under in vitro conditions, bovine granulosa cells subjected to thermal stress release EVs with different miRNA cargo have the potential to shuttle protective messages to recipient cells inducing thermotolerance against subsequent HS [20]. Therefore, unveiling the ovarian FF-EV cargoes in response to environmental thermal stress conditions in vivo will aid to elucidate the EV-mediated molecular response and potential impact on follicular development and oocyte growth. Here we aimed to investigate the changes in FF-EV-coupled miRNAs in beef cows when comparing winter and summer seasons. The deviation in EV-miRNA profiles in summer compared to the winter season may in part explain the abnormal ovarian function, follicular development, and infertility-associated problems due to climate change-induced thermal stress in dairy and beef animals [41, 42].

Among the top 20 highly abundant miRNAs, 18 were commonly expressed in both groups including miR-148a and miR-99a, the top highly expressed miRNAs in the FF-EVs with no significant differences between the groups. Similarly, miR-148 was reported as the top expressed miRNA in FF-EVs from large and small follicles in goats [43]. In human FFs, miR-99a was specifically enriched in the FF-EVs and considered as a regulator of the ovarian follicle developmental process to include meiosis resumption [44]. In another study, the expression of miR-148a and miR-99a in FF-EVs was highly correlated with IVF outcomes, specifically day-3 embryo quality [45]. The detection of these candidate miRNAs at a higher level in both seasons indicates their potential housekeeping role in ovarian function. In the same top 20 miRNAs list, miR-10a and miR-10b were expressed amid both groups and were significantly upregulated in the SUM compared to the WIN group. Both miRNAs belong to the conserved mir-10 family and are known to play key roles in inducing apoptosis and repressing cell proliferation in ovarian GCs through suppressing the brain-derived neurotrophic factor (BDNF) and TGF-β pathways [46]. Additionally, miR-10a has been reported to promote human GC tumor development by targeting PTEN-AKT/Wnt pathways [47] and regulating lipid metabolism and steroid hormone synthesis in sheep GCs [48]. Previously, it has been validated that miR-10a directly recognizes and targets the *BCL6* transcript and negatively regulates its expression leading to cellular apoptosis [49]. In correlation with HS, miR-10a was suggested to be involved in the stress response pathway regulation via its repression of a number of p53/Rb networks’ key genes [50]. Therefore, our results indicate the negative impact of seasonal HS on ovarian function is mediated by the enrichment of miR-10a and miR-10b in EVs released in response to thermal stress. Another interesting miRNA from the same top 20 miRNAs list was miR-26a which exhibited a significant downregulation in the SUM compared to the WIN group. A similar study in heat-stressed Holstein cows showed that the expression of miR-26a was downregulated in serum and found to be involved in stress and immune response-related processes [51]. Moreover, a HS-induced increase in corticosterone hormone is similarly found to be associated with the reduction in miR-26a in rat serum EVs [52]. In addition, it has been confirmed that miR-26a targets Exh2 and plays a critical role in regulating apoptosis in mouse ovarian GCs [53].

In the current study, we found a total of 24 DE-miRNAs in the FF-EVs between the SUM and WIN groups. Among the upregulated miRNAs, four (miR-19a, miR-19b, miR-30a-5p, and miR-200a) were commonly identified as highly expressed in the serum of heat-stressed Holstein cows [51]. In another study, miR-19a and miR-19b were identified among the circulatory miRNAs that were highly expressed in lactating Holstein cows under summer HS conditions and were correlated with functions governing responses to stress and oxidative damage [54]. Moreover, heat shock during in vitro maturation of bovine oocytes was shown to increase the expression of miR-19b in embryos, indicating a carryover impact of HS on miRNAs in the cellular response [55]. In humans, miR-19b has been reported as an inhibitor of GC proliferation by directly targeting *IGF-1* and the reduction in its expression could reverse the results [56] suggesting the important regulatory and specific role of miR-19b in GCs and could explain the oocyte quality and fertility reduction during the summer season. The top-upregulated miRNA in the SUM group was miR-184. This miRNA is associated with an inhibitory effect on GC estradiol production [57] and was found to be increased in bovine GCs at day 7 compared to day 3 of the estrous cycle [58].

On the other hand, a cluster of eight miRNAs was downregulated in the SUM FF-EVs compared to the WIN group including miR-181a and miR-1246. Both miRNAs showed a clear reduction in buffalo GCs cultured under thermal stress conditions [59] and are known to be involved in stress and immune responses [51, 60]. Inhibition of miR-181a expression suppresses apoptosis and ROS production in human chondrocyte cells [61] and reduces heat stress damage through the upregulation of antioxidant-related genes and the downregulation of apoptotic genes in bovine peripheral blood mononuclear cells [62]. Contrary to our findings, miR-1246 was found to be highly enriched in the serum of dairy cows under environmental HS [51, 54], as well as, in the GC-released EVs under in vitro elevated culture temperature [20]. However, the expression of miR-1246 in correlation with HS seems to be time-dependent, as in cattle and buffalo fibroblast cells, the expression of miR-1246 declined immediately after HS and then increased gradually during the recovery period post-HS [60]. This discrepancy in the differential expression results of EV-miRNA-1246 might be associated with the timing of exposure and recovery to thermal stress. Taken together, our data revealed that seasonal HS can induce the enrichment of FF-EV-coupled miRNAs, with a potential negative impact on ovarian function (i.e. miR-10 family) and the depletion of candidate miRNAs (i.e. miR-26a) with the potential beneficial role in ovarian physiology.

Although EV miRNA abundance is altered under suboptimal physiological conditions to include HS, little is known about the mechanism associated with their sorting into EVs versus their cellular retention. Recently, the regulatory role of RBPs in the packaging of particular RNA molecules into EVs following the recognition and binding of specific sequence motifs is emerging among different models [63, 64]. Sequence motif analysis for EV-coupled miRNAs enriched in SUM samples revealed two specific motifs appearing on 13 out of the 16 upregulated miRNAs and recognized by specific RBPs. One of those motifs was found to be potentially recognized and bound by Y-box binding proteins (YBX1 and YBX2). These proteins are members of a large family of proteins with the cold shock domain that play roles in several cellular processes including proliferation and stress response [65, 66]. A study by Guarino et al*.* reported that oxidative stress enhances the secretion of YBX1 protein from stressed cells which significantly inhibits proliferation and leads to cell cycle arrest in receiving cells [67]. Y-box proteins have been identified as the main components in the formation of ribonucleoprotein particles with the different types of RNA including mRNA and miRNA [68] and play a role in sorting these RNA molecules into EVs [69]. Regarding miRNAs, it has been evidenced that YBX1 binds to and is required for the sorting of specific miRNAs into EVs released from the HEK293T human cell line [70]. Another interesting RBP that matched with the same sequence motif was RBM42, which together with the hnRNP K is part of the stress granules. Under stress conditions, both proteins co-localize and interact to form cytoplasmic foci in order to maintain cellular ATP levels [71]. The expression of these proteins in correlation to stress conditions, as well as, their matching of common sequence motifs among the upregulated miRNAs in the SUM group could explain a potential mechanism involved in sorting and releasing of these FF-EV-miRNAs under HS conditions. However, further studies are required to specifically investigate the role of miRNA motifs and RBPs in regulating the sorting and packaging of stress associated miRNAs into EVs and the role that this interplay drives on follicular cells stress response and survival under various environmental and physiological suboptimal conditions.

## Conclusions

Overall, the current study revealed that FF-EV-miRNA profiles can be explored to investigate the follicular level response of cows to seasonal changes and this might partially explain altered ovarian physiology, follicular development, and infertility-associated issues resulting from climate change-induced seasonal thermal stress in the dairy and beef industry. Based on the differentially expressed miRNAs, we suggest that the negative impact of HS on ovarian function is mediated by abnormal alterations in EV-coupled molecular signaling within the follicular microenvironment, as shown by the contrasting finding when comparing summer and winter EV-miRNAs. However, further studies are needed to confirm and investigate the exact role of these EV-miRNAs concerning seasonal effects and fertility. The current study also identified potential targets of future therapeutic and managerial intervention to tackle the negative impact of environmental thermal stress in cattle with potential translation to seasonal human infertility problems.

## Supplementary Information

**Additional file 1: Table S1.** Summary of sequence reads mapped to the bovine reference genome and annotated against bovine miRNAs listed in the mirBase database. **Table S2.** A complete list of all expressed miRNAs indicated as the mean of TMM-adjusted Counts Per Million (CPM) value. **Table S3.** KEGG pathway analysis for genes targted by the differentially expressed miRNAs in SUM vs. WIN group. **Table S4.** Biological process (BP) analysis for genes targted by the differentially expressed miRNAs in SUM vs. WIN group.

## Data Availability

The raw FASTQ files and processed CSV files of the miRNA sequencing data have been deposited in the NCBI’s Gene Expression Omnibus (GEO) repository with the accession number GSE221198. All parameters related to EV experiments are available on the EV-TRACK knowledgebase (EV-TRACK ID: EV220402).
